# Severe hyperlactatemia with normal base excess: a quantitative analysis using conventional and Stewart approaches

**DOI:** 10.1186/cc6896

**Published:** 2008-05-08

**Authors:** Graciela Tuhay, María Carolina Pein, Fabio Daniel Masevicius, Daniela Olmos Kutscherauer, Arnaldo Dubin

**Affiliations:** 1 Servicio de Terapia Intensiva, Sanatorio Otamendi y Miroli, Buenos Aires, Argentina

## Abstract

**Introduction:**

Critically ill patients might present complex acid–base disorders, even when the pH, PCO_2_, [HCO_3_^-^], and base excess ([BE]) levels are normal. Our hypothesis was that the acidifying effect of severe hyperlactatemia is frequently masked by alkalinizing processes that normalize the [BE]. The goal of the present study was therefore to quantify these disorders using both Stewart and conventional approaches.

**Methods:**

A total of 1,592 consecutive patients were prospectively evaluated on intensive care unit admission. Patients with severe hyperlactatemia (lactate level ≥ 4.0 mmol/l) were grouped according to low or normal [BE] values (<-3 mmol/l or >-3 mmol/l).

**Results:**

Severe hyperlactatemia was present in 168 of the patients (11%). One hundred and thirty-four (80%) patients had low [BE] levels while 34 (20%) patients did not. Shock was more frequently present in the low [BE] group (46% versus 24%, *P *= 0.02) and chronic obstructive pulmonary disease in the normal [BE] group (38% versus 4%, *P *< 0.0001). Levels of lactate were slightly higher in patients with low [BE] (6.4 ± 2.4 mmol/l versus 5.6 ± 2.1 mmol/l, *P *= 0.08). According to our study design, the pH, [HCO_3_^-^], and strong-ion difference values were lower in patients with low [BE]. Patients with normal [BE] had lower plasma [Cl^-^] (100 ± 6 mmol/l versus 107 ± 5 mmol/l, *P *< 0.0001) and higher differences between the changes in anion gap and [HCO_3_^-^] (5 ± 6 mmol/l versus 1 ± 4 mmol/l, *P *< 0.0001).

**Conclusion:**

Critically ill patients may present severe hyperlactatemia with normal values of pH, [HCO_3_^-^], and [BE] as a result of associated hypochloremic alkalosis.

## Introduction

Metabolic acidosis of hypoxic states or anaerobic exercise has been traditionally explained by lactate production. Nevertheless, there is biochemical evidence that lactate production does not cause acidosis, but retards its development [1,2]. During anaerobic metabolism, protons derived from ATP hydrolysis that cannot be reutilized in oxidative phosphorylation might be the actual explanation for metabolic acidosis [1,2]. Nevertheless, there is some evidence showing that aerobic lactate production (that is, during catecholamine administration) is associated with metabolic acidosis [3,4].

Whichever mechanism produces acidosis, increased lactate production coincides with cellular acidosis, and remains a good indirect marker for cell metabolic conditions that induce metabolic acidosis. Many studies have consequently established the use of blood-lactate levels as a diagnostic, therapeutic, and prognostic marker of tissue hypoxia in circulatory shock [5]. In addition, lactic acidosis is the most frequent cause of metabolic acidosis [6] and one of the most common metabolic abnormalities in critically ill patients [5]. Moreover, Gunnerson and colleagues demonstrated a higher mortality in critically ill patients with lactic acidosis than in patients with hyperchloremic acidosis [7]. For a correct diagnostic and prognostic evaluation of critically ill patients, therefore, severe metabolic acid–base disorders such as lactic acidosis must be identified.

Lactic acidosis is primarily suspected because of the presence of metabolic acidosis. Nevertheless, [HCO_3_^-^] and base excess ([BE]) levels might be normal despite the presence of hyperlactatemia, as a result of simultaneous alkalinizing processes. Accordingly, Fencl and colleagues showed that, in 152 critically ill patients, Stewart's approach could detect metabolic acidosis in some patients with normal [HCO_3_^-^] and [BE] levels [8]. In those patients, the metabolic acidosis with a low strong-ion difference ([SID]) was counterbalanced by alkalinizing processes [8].

Although the lack of correlation of hyperlactatemia with pH, [HCO_3_^-^], and [BE] values has been previously reported, these reports have not used a systematic approach to understand the underlying metabolic acid–base disorders [9-13]. The objective of the present investigation was to study a large series of critically ill patients with high lactate levels and to quantitatively analyze the presence of alkalinizing processes that might neutralize the decrease of [BE], and thus occult metabolic disorders. Our hypothesis was that the metabolic acidosis associated with hyperlactatemia could be frequently hidden by the effect of alkalinizing processes that neutralize [BE].

## Methods and materials

### Participants

A prospective observational study was performed in a university-affiliated hospital intensive care unit (ICU). A total of 1,592 consecutive patients were immediately evaluated on ICU admission during a period of 3 years from 1 March 2004 to 28 February 2007. Each patient with severe hyperlactatemia (lactate level ≥ 4.0 mmol/l) was included.

This study was approved by the Institutional Ethics Committee. Since standard procedures were applied in the diagnostic management, informed consent from patients was waived. The patients participating in this study are part of a large database, and some of them have been included in a previous publication [14].

### Measurements

On ICU admission, demographic data (age, gender), type of admission (surgical or medical), presence of shock [15], previous history of chronic obstructive pulmonary disease, administration of diuretics, volume and type of fluid administered before ICU admission, and the use of mechanical ventilation were recorded. The Acute Physiologic and Chronic Health Evaluation II score [16], the predicted risk of mortality, the Sepsis-related Organ Failure Assessment score [17], and the McCabe score [18] were calculated.

Arterial blood samples were analyzed for gases (AVL OMNI 9; Roche Diagnostics, Graz, Austria), and for the concentrations [Na], [K] and [Cl^-^] (selective electrode ion, AEROSET; Abbott Laboratories, Abbott Park, IL, USA), [Ca] (selective electrode ion, AVL OMNI 9; Roche Diagnostics), and [Mg] (Arsenazo dye/magnesium complex), [albumin] (bromcresol-sulfonphthaleinyl), inorganic phosphate [P_i_^-^] (molybdate–vanadate), and [lactate] (selective electrode ion, AVL OMNI 9).

### Calculated values

The values for [HCO_3_^-^] and [BE] (extracellular) were calculated by means of the Henderson–Hasselbalch [19,20] and Van Slyke equations [21,22], respectively.

The anion gap [AG] was calculated as [23]:

[AG] = ([Na^+^] + [K^+^]) - ([Cl^-^] + [HCO_3_^-^])

The [AG] was then corrected for the effect of abnormal albumin concentration (in g/l) [24]:

[AG]_corrected _(mmol/l) = [AG]_observed _+ 0.25 × ([normal albumin] - [observed albumin])

The effective [SID] was calculated as [8]:

[SID]_effective _= [HCO_3_^-^] + [albumin^-^] + [P_i_^-^]

The [albumin^-^] and [P_i_^-^] (mmol/l) values were calculated from the measured [albumin] (g/l), [P_i_] (mmol/l), and pH levels as [8]:

[albumin^-^] = [albumin] × (0.123 × pH - 0.631)

[P_i_^-^] = [P_i_] × (0.309 × pH - 0.469)

The apparent [SID] was calculated as [8]:

[SID]_apparent _= [Na^+^] + [K^+^] + [Ca^2+^] + [Mg^2+^] - [Cl^-^]

The strong ion gap ([SIG]) is composed of strong anions other than [Cl^-^] (lactate, ketoacids and other organic anions, sulfate), and was calculated as [8]:

[SIG] = [SID]_apparent _- [SID]_effective_

The total concentration of plasma nonvolatile buffers ([A_tot_^-^]) was calculated as [25]:

[A_tot_^-^] = [albumin^-^] + [P_i_^-^]

Differences between the changes in [AG]_corrected _and [HCO_3_^-^] (Δ[AG]_corrected _- Δ[HCO_3_^-^]) and between the changes in [AG]_corrected _and [BE] (Δ[AG]_corrected _- Δ[BE]) were calculated.

The [Cl^-^] and [SIG] levels were adjusted to water excess/deficit by multiplying the observed value by a correcting factor ([Na^+^]_normal_/[Na^+^]_observed_) [8].

### Data analysis

Patients were separated into two groups according to [BE] < -3 mmol/l or [BE] > -3 mmol/l. Data are expressed as the mean ± standard deviation or the median (interquartile range, 0.25 to 0.75), as appropriate. The data were analyzed with the Student *t *test and the Mann-Whitney *U *test for unpaired samples, and with the chi-square test for categorical variables. *P *< 0.05 was considered statistically significant.

## Results

Severe hyperlactatemia was present in 168 of the patients (11%). One-hundred and thirty-four (80%) patients had low [BE] values while 34 (20%) did not.

Clinic, epidemiologic, and outcome data are presented in Table 1. Both groups had similar values of the Acute Physiologic and Chronic Health Evaluation II score, the predicted and actual mortality, the Sepsis-related Organ Failure Assessment score, and the McCabe score. Patients with low [BE] were more frequently associated with shock and surgical admission. Chronic obstructive pulmonary disease and medical admission were more commonly found in patients with normal [BE].

**Table 1:** Clinical, epidemiological and outcome data

	Low base excess group	Normal base excess group	*P *value
*n *(%)	134 (80)	34 (20)	
Age (years)	63 ± 19	68 ± 14	0.15
Gender, male (%)	49	48	0.88
APACHE II score	16 ± 11	15 ± 8	0.41
APACHE II predicted mortality (%)	31 ± 27	26 ± 21	0.33
Actual mortality (%)	19	17	0.53
McCabe score	1.8 ± 0.7	1.8 ± 0.8	0.62
SOFA score	5 ± 5	4 ± 3	0.16
Medical/surgical admission (%)	47	82	0.0002
Type of surgical admission			
Elective (%)	34	15	<0.03
Emergency (%)	15	3	0.06
Trauma (%)	3	0	0.31
Transferred from			
Emergency department (%)	40	71	<0.002
General ward (%)	11	3	0.14
Time to intensive care unit admission (hours)	3 (1 to 5)	2 (2 to 5)	0.90
Shock (%)	46	24	0.02
Chronic obstructive pulmonary disease (%)	4	38	<0.0001
Sepsis (%)	23	21	0.75
Stroke	15	6	0.09
Mechanical ventilation (%)	40	35	0.22
Total bilirubin (mg%)	1.1 ± 1.2	1.1 ± 1.7	0.65
Plasma urea (mg%)	46 ± 31	54 ± 32	0.24
Plasma creatinine (mg%)	1.3 ± 0.7	1.1 ± 0.5	0.26

APACHE, Acute Physiologic and Chronic Health Evaluation; SOFA, Sepsis-related Organ Failure Assessment.

Patients with low [BE] received more saline solution before ICU admission (1,000 (500 to 2,000) versus 0 (0 to 500) ml, *P *= 0.0004). There were no differences in the volume of Ringer-lactate solution received (0 (0 to 1,000) versus 0 (0 to 0) ml, *P *= 0.18). Twenty-one percent of the patients in each group received diuretics before ICU admission (*P *= 0.97).

**Figure 1:**
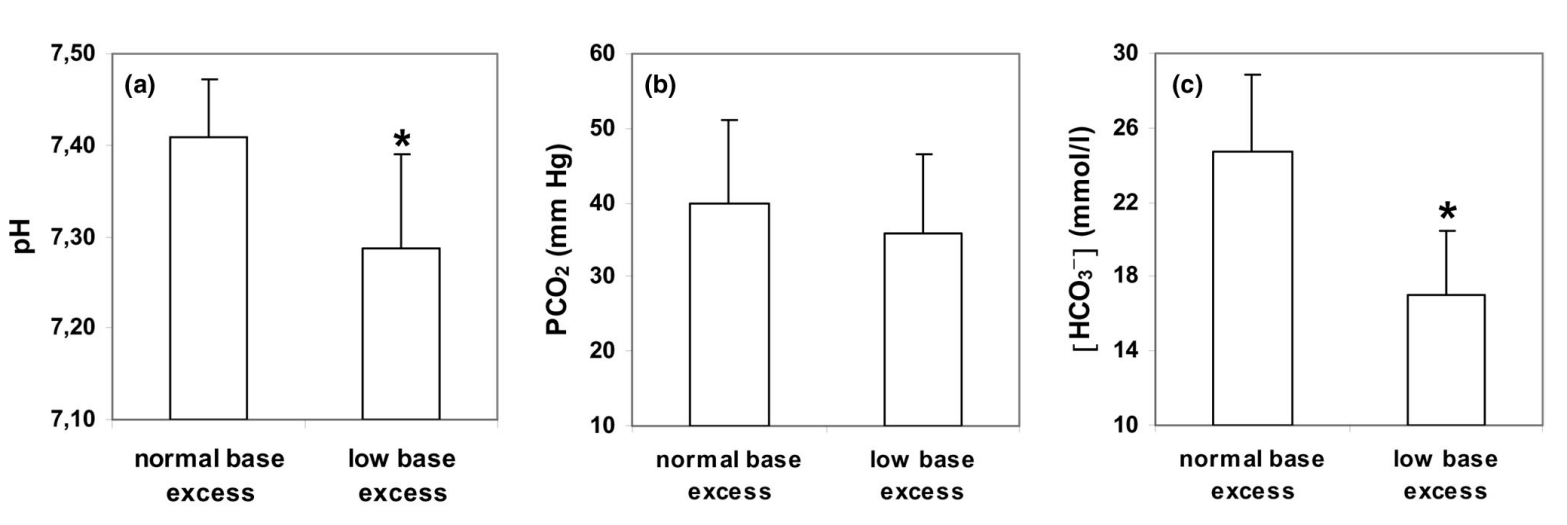
Arterial pH, PCO_2_, and bicarbonate levels in patients with severe hyperlactatemia. Values for **(a) **arterial pH, **(b) **PCO_2_, and **(c) **bicarbonate ([HCO_3_^-^]) in patients with severe hyperlactatemia, with normal or low base excess. *P < 0.05 versus the other group.

**Figure 2:**
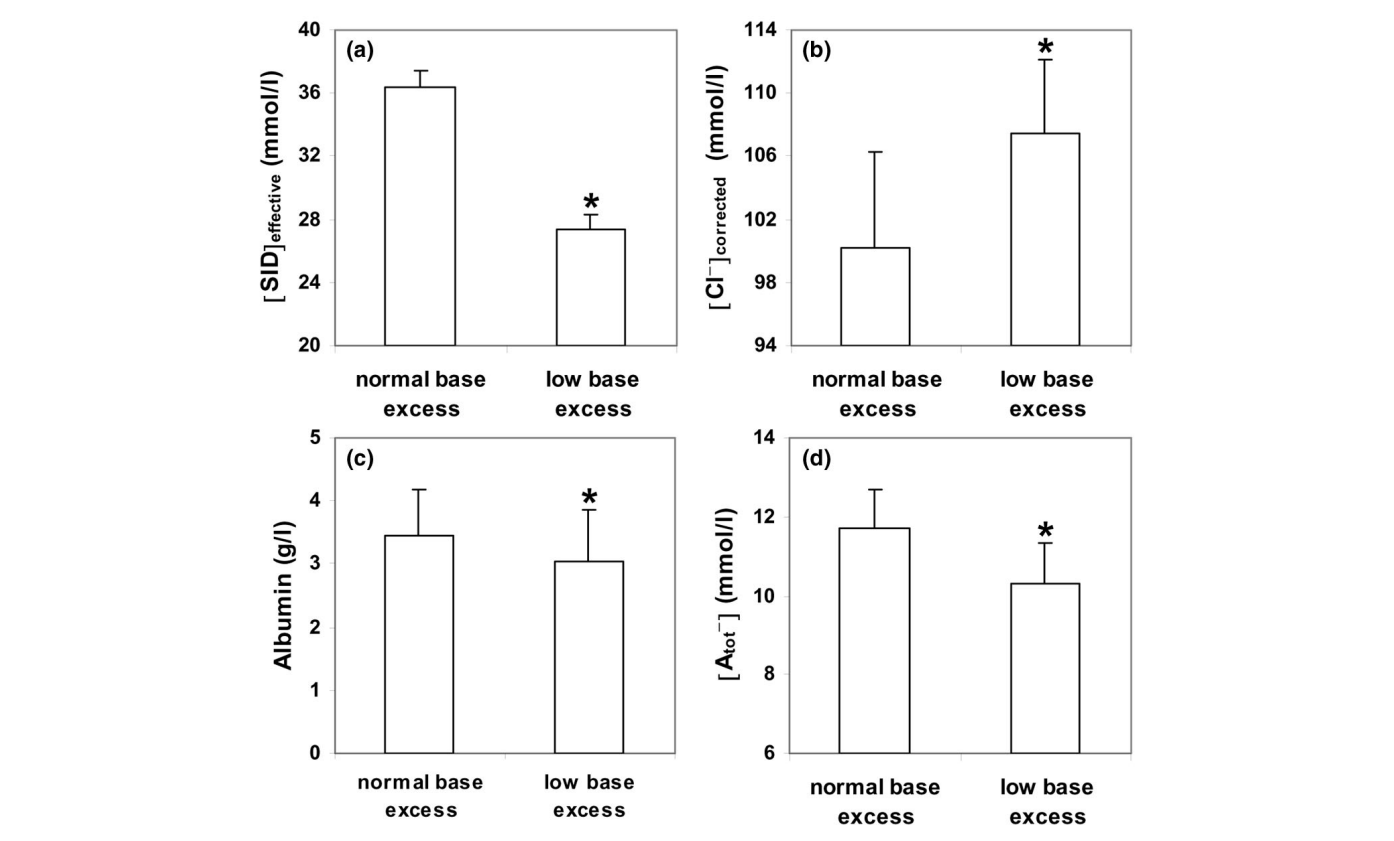
Effective strong-ion difference, sodium-corrected chloride, albumin, and nonvolatile weak acid levels in severe hyperlactatemia patients. Values for **(a) **the effective strong-ion difference ([SID]_effective_), **(b) **sodium-corrected chloride levels ([Cl^-^]_corrected_), **(c) **the albumin concentration, and **(d) **nonvolatile weak acid ([A_tot_^-^]) levels in patients with severe hyperlactatemia, with normal or low base excess. *P < 0.05 versus the other group.

Levels of lactate were slightly higher in patients with low [BE] (6.4 ± 2.4 mmol/l versus 5.6 ± 2.1 mmol/l, *P *= 0.08). According to the study design, the pH, [HCO_3_^-^], and [SID] levels were lower in patients with low [BE] (Figures 1 and 2). Patients with normal [BE] had lower [Cl^-^]_corrected _(Figure 2) and higher Δ[AG]_corrected _- [HCO_3_^-^] and Δ[AG]_corrected _- Δ[BE] values (5 ± 6 mmol/l versus 1 ± 4 mmol/l and 3 ± 6 mmol/l versus 4 ± 4 mmol/l, respectively; *P *< 0.0001 for both). These patients also had levels of [AG]_corrected _and [SIG]_corrected _that were slightly lower (21 ± 5 mmol/l versus 23 ± 5 mmol/l, *P *= 0.07 and 9 ± 5 mmol/l versus 11 ± 5 mmol/l, *P *< 0.05, respectively). The [albumin] and [A_tot_^-^] values were lower in patients with low [BE] (Figure 2).

Since the normal reference intervals for plasma [Cl^-^]_corrected _are 103 to 111 mmol/l in our laboratory, most of the patients in the normal [BE] group had absolute hypochloremia (65% of the patients). Conversely, most of the patients in the low [BE] group had normal [Cl^-^]_corrected _levels (90% of the patients).

The normal [BE], [HCO_3_^-^], and [SID] levels were almost completely explained by the alkalinizing effect of hypochloremia. A quantitative analysis shows that the differences in mean [BE], [HCO_3_^-^], and [SID] values between both groups were about 8 mmol/l (lower in the low [BE] group) while the difference in the [Cl^-^] level was 7 mmol/l.

## Discussion

The main finding of the present study was that 20% of the patients with severe hyperlactatemia showed normal pH, [HCO_3_^-^], [BE], and [SID] levels because of the simultaneous presence of hypochloremic metabolic alkalosis.

Madias reported previously that in lactic acidosis the increase in the [AG] might be occasionally greater than the decrease in the corresponding [HCO_3_^-^] [26]. This finding indicates the diagnosis of a mixed metabolic disorder [27]. The actual incidence of mixed metabolic disorder in patients with severe hyperlactatemia has not been previously described. To our knowledge, the present study is the first to systematically address this issue and quantify the underlying metabolic acid–base.

Acid–base disorders might be characterized by different methods. First, by a traditional approach in which the metabolic component of acid–base physiology is assessed by analysis of [HCO_3_^-^] levels [27]. The evaluation of the metabolic component might be further completed by the inclusion of [BE] [28]. Despite considerable argument about which of these parameters is better [29-32], both are usually employed in clinical practice and their calculations are included in all blood-gas analyzers. The [AG] constitutes an additional diagnostic contribution [23], although hypoalbuminemia might decrease the usefulness of this parameter. For this reason, many researchers have recommended adjusting the [AG] for the albumin level [25,33-37].

An alternative approach is the application of basic physicochemical principles of aqueous solutions to blood. Stewart identified variables that primarily and independently of one another determine the pH [38]: PCO_2_, the [SID] (that is, the difference between the sums of all the strong cations and all the strong anions), and the [A_tot_^-^]. Using this approach, Fencl and colleagues have shown that the traditional analysis frequently failed to identify severe disturbances such as metabolic acidosis [8]. In that study, a low [SID] was undetected through changes in [BE] because the low [SID] acidosis was masked by the alkalinizing effect of hypoalbuminemia present in all patients.

As we previously shown [14], however, the combined use of [HCO_3_^-^], [BE], and [AG]_corrected _allowed the same acid–base diagnosis. Accordingly, in the group with normal [BE], normal values of pH, [HCO_3_^-^], and [BE] matched with a normal [SID]. The diagnosis of mixed metabolic acidosis and alkalosis was performed by the presence of positive Δ[AG]_corrected _- Δ[HCO_3_^-^] and Δ[AG]_corrected _- Δ[BE] levels [27] in the traditional analysis, and by increased [SIG] and low [Cl^-^]_corrected _values in Stewart's approach [8]. Our data reinforce the concept that acid–base analysis only based on pH, [HCO_3_^-^], and [BE] values might be frequently misleading. An adequate diagnosis should rely on a more comprehensive approach that might include the use of [AG]_corrected _or chloride levels.

Different to previous studies in which hypoalbuminemia was the confounding factor in the interpretation of acid–base data [8], the alkalinizing factor in the present study was hypochloremia. Albuminemia and the [A_tot_^-^] value were lower in patients with low [BE] – which might be due to the presence of shock, a condition that increases extravascular albumin losses [39]. McAuliffe and colleagues described 'primary hypoproteinemic alkalosis' in hypoalbuminemic ICU patients with positive [BE] and elevated [HCO_3_^-^] levels [40]. Nevertheless, the actual role of hypoalbuminemia to produce metabolic alkalosis has been recently challenged [14,40]. We previously could only detect one patient fulfilling the criteria of primary hypoproteinemic alkalosis among 700 hypoalbuminemic patients [14]. Wilkes showed that the loss of weak acid secondary to hypoproteinemia is compensated by a renal-mediated increase in [Cl^-^], so the [SID] decreases without changes in pH [41].

The presence of hypochloremic metabolic alkalosis in patients with normal [BE] can be related to the high number of patients with chronic obstructive pulmonary disease. In these patients, hypochloremia is the consequence of an appropriate kidney response to chronic respiratory acidosis [42]. Despite the prior administration of diuretics being similar both groups, in some patients the diuretics might have contributed to the development of hypochloremia.

Although most of the patients in the normal [BE] group had absolute hypochloremia and most of the patients in the low [BE] group had normal [Cl^-^] levels, a mechanism other than hypochloremic alkalosis might have contributed to the differences in [Cl^-^] between both groups. Since shock was more frequently present in patients with low [BE], these patients received more aggressive fluid resuscitation before ICU admission. Consequently, a subtle component of hyperchloremic metabolic acidosis might be present in this group [43].

In a study of patients admitted to the ICU after cardiac arrest, lactic acidosis was the most frequent disorder. Its effects on the pH, however, were attenuated by the presence of a metabolic alkalosis caused by hypochloremia and hypoalbuminemia. The authors explained these findings by extravascular passage of albumin, and pre-existing disease [44].

The presence of metabolic acidosis in critical patients has prognostic implications. Gunnerson and colleagues recently demonstrated that patients with metabolic acidosis ([BE] < -2 mmol/l) had a higher mortality rate than those without this disorder (45% versus 25%). Similarly, patients with lactic acidosis had higher mortality than those with hyperchloremic acidosis (56% versus 29%) [7]. Nevertheless, it is not clear whether differences in outcome are dependent on the process that produces metabolic acidosis or on the acidosis itself.

Although our two groups of patients had quite different pH values, their mortality was not different. A possible explanation for this observation might be that the severity of critical illness, as evaluated by the Acute Physiologic and Chronic Health Evaluation II and Sepsis-related Organ Failure Assessment scores, was similar in both groups. Recent data showed that metabolic acid–base variables had a poor discriminating ability for predicting mortality in a general ICU population. Areas under receiver operating characteristic curves for acid–base parameters were significantly lower than that of the Sepsis-related Organ Failure Assessment score [14]. Nevertheless, a higher number of patients are required to confirm that the presence of acidemia itself does not worsen the outcome.

The present study has some limitations. The study is observational, aimed at describing the incidence of severe hyperlactatemia with normal [BE] and quantifying its underlying acid–base alterations. Nevertheless, patients were only evaluated on ICU admission. Serial measurements might have allowed a more comprehensive understanding of the acid–base disorders and allowed better insights into the mechanisms of acid–base disorders.

## Conclusion

Our results suggest that 20% of critically ill patients have severe hyperlactatemia with normal pH, [HCO_3_^-^], and [BE] levels because of a concomitant presence of hypochloremic alkalosis. Both the conventional and Stewart approaches allow the identification of this mixed metabolic disorder. The results also suggest the evaluation of plasma [Cl^-^] and Δ[AG]_corrected _- Δ[HCO_3_^-^] levels should always be considered for a correct diagnosis of acid–base disorder.

## Key messages

• Twenty percent of critically ill patients have severe hyperlactatemia with normal pH, [HCO_3_^-^], and [BE] levels because of a concomitant presence of hypochloremic alkalosis.

• As previously shown, both the conventional and Stewart approaches allow the correct identification of mixed metabolic acidosis and alkalosis.

• The evaluation of plasma chloride and the difference between the changes in the anion gap and bicarbonate should always be considered for a correct diagnosis of acid–base disorders.

## Abbreviations

[AG] = anion gap; [A_tot_^-^] = total concentration of plasma nonvolatile buffers; [BE] = base excess; [HCO_3_^-^] = bicarbonate concentration; PCO_2 _= partial pressure of carbon dioxide; [P_i_] = inorganic phosphate concentration; [SID] = strong-ion difference; ICU = intensive care unit; [SIG] = strong-ion gap.

## Competing interests

The authors declare that they have no competing interests.

## Authors' contributions

GT and MCP mainly contributed to the conception and design of the study. GT, MCP, FDM and DOK performed acquisition of data, and contributed to the analysis and interpretation of data. AD drafted the manuscript and performed the statistical analysis. All authors read and approved the final manuscript.
